# Orphan gene *BR2* positively regulates bolting resistance through the vernalization pathway in Chinese cabbage

**DOI:** 10.1093/hr/uhae216

**Published:** 2024-07-30

**Authors:** Ye Zu, Mingliang Jiang, Zongxiang Zhan, Xiaonan Li, Zhongyun Piao

**Affiliations:** Molecular Biology of Vegetable Laboratory, College of Horticulture, Shenyang Agricultural University, Shenyang 110866, China; School of Agriculture, Jilin Agricultural Science and Technology University, Jilin 132101, China; Molecular Biology of Vegetable Laboratory, College of Horticulture, Shenyang Agricultural University, Shenyang 110866, China; Molecular Biology of Vegetable Laboratory, College of Horticulture, Shenyang Agricultural University, Shenyang 110866, China; Molecular Biology of Vegetable Laboratory, College of Horticulture, Shenyang Agricultural University, Shenyang 110866, China

## Abstract

Orphan genes (*OGs*) are unique to the specific species or lineage, and whose homologous sequences cannot be found in other species or lineages. Furthermore, these genes lack recognizable domains or functional motifs, which make their characterization difficult. Here, we identified a *Brassica rapa*  *OG* named *BOLTING RESISTANCE 2* (*BR2*) that could positively modulate bolting resistance. The expression of *BR2* was developmentally regulated and the *BR2* protein was localized to the cell membrane. *BR2* overexpression not only markedly delayed flowering time in *Arabidopsis* transgenic plants, but substantially affected the development of leaves and flower organs. Flowering repressor *AtFLC* gene was significantly up-regulated transcribed in *Arabidopsis BR2* overexpression lines, while *AtFT* and *AtSOC1* expression was decreased. In addition, the *BR2* expression was enhanced in bolting-resistant type Chinese cabbage and was reduced in non-resistant type. Moreover, chilling stress inhibited the *BR2* expression levels. Overexpression of *BR2* also delayed flowering time in Chinese cabbage. In vernalized Chinese cabbage *BR2* overexpression plants, *BrVIN3.b* and *BrFRI* were significantly down-regulated, while *BrFLC5* was substantially up-regulated. Key floral factors, including three *BrSOC1s*, two *BrLFYs*, and four *BrFTs* were down-regulated. The expression changes of these key genes were consistent with the delayed flowering phenotype of Chinese cabbage *BR2* overexpressing plants. Thus, we predicted that *BR2* may predominantly function *via* the vernalization pathway. Our findings propose that the *OG*  *BR2* acts as a novel modulator of flowering time in Chinese cabbage, which provides a new insight on the breeding of varieties that are resistant to bolting.

## Introduction

Orphan genes (*OGs*) are unique genes that appear in the long-term evolution of species and have no significant sequence similarity in other species [1, 2]. With the continuous advancements in genome sequencing technology, *OGs* have been identified in different species and proved to function as mediators during metabolite synthesis [3–6], biotic stress response [7–12], abiotic stress response [13–15], species-specific characteristics, or growth and development regulation [16–20]. In *Cucurbitaceae*, *OGs* crucially linked with various pathways, including crop domestication, male sterility regulation, and environmental adaptation [21]. *Arabidopsis thaliana*  *OGs* had a significant response to environmental stimuli [22]. Furthermore, in domesticated cowpea, *OGs* have also been associated with ecoclimatic-mediated selections and drought adaptations [14]. Jiang *et al*. identified 529 *Brassica rapa*  *OGs* in silico and constructed a 43 *B. rapa OG*s overexpression library in *A. thaliana* [6, 23]. Most of these *B. rapa*  *OGs* affected soluble sugar metabolism [6]. The functions of these *OGs* indicate their application potential in breeding, which can improve plant stress resistance and quality. Although *OGs* are associated with stress responses as well as their high specificity of expressions having been extensively studied, the functions of *OGs* in the *B. rapa* require further exploration.

In plants, flowering is a significant developmental event that modulates the transition from vegetative to reproductive growth. Currently, five genetically defined pathways are known to regulate floral transition, mainly including the photoperiod, age, vernalization, autonomous, and gibberellin (GA) pathways [24, 25]. The two crucial vernalization pathway genes are *FLOWERING LOCUS C* (*FLC*) and *FRIGIDA* (*FRI*) [26, 27]. *FLC* is a transcription factor of MADS-box that can suppress flower transformation, whereas *FRI* acts upstream of *FLC* and positively inhibits flowering [26]. Besides *FRI*, *VERNALIZATION INSENSITIVE 3* (*VIN3*) is also an important gene that regulates *FLC* [28, 29]. The antisense transcripts of *FLC* include selective polyadenylation of RNA binding proteins FLOWERING CONTROL LOCUS A (FCA) and *COOLAIR*, through which *FLC* transcription expresses silence mechanism when it is warm. Proximal polyadenylation is associated with *FLC*’s histone demethylation and chromatin alterations induced by PRC2 [30–32]. In addition, *FLC* also markedly suppresses genes that induce flowering in plants, including *FLOWERING LOCUS T* (*FT*), *SUPPRESSOR OF OVEREXPRESSION OF CO1* (*SOC1*), and *LEAFY* (*LFY*) [33]. Overexpression of *B. rapa FLC* homologs in *Arabidopsis* delayed flowering for nearly 4–7 weeks [34]. However, *FLC*’s cold-sensing mechanism in *B. rapa* has not been comprehensively studied.

Chinese cabbage (*B. rapa* ssp. *pekinensis*) is one of the leafy vegetables with a wide planting area and the highest total yield in China. However, premature bolting leading to non-heading is a major problem in Chinese cabbage production, which severely affects the yield, especially during autumn at high latitudes and high-altitude regions, as well as in spring in northern areas of China [35, 36]. Therefore, breeding bolting-resistant Chinese cabbage varieties has always been the primary concern of breeders. The genome triplication event of *B. rapa* has produced several flowering-related genes, which regulate mechanisms much more complicated than that of *A. thaliana* [37]. Altogether, four *FLC* paralogues (*BrFLC1*, *BrFLC2*, *BrFLC4*, *BrFLC5*) have been cloned in *B. rapa* [38]. Furthermore, *BrFLC2* has been indicated as a primary regulator gene of flowering time and vernalization response QTL in *B. rapa* [39, 40]. Moreover, natural splicing mutation at *BrFLC1* intron site has been markedly linked with altered flowering time in *B. rapa* [41]. Previous study indicates that *BrFLC5* (*Bra022771*) is expressed at lower levels than two other *BrFLC* genes (*BrFLC1*, *Bra009055* and *BrFLC2*, *Bra028599*) indicating that *BrFLC5* is more conducive to cultivating varieties in *B. rapa* which are resistant to bolting [42]. In addition, a histone H4 protein encoded by *BrHIS4.A04* (*Bra035673*) has previously been shown to inhibit drought-mediated bolting in Chinese cabbage by attenuating the flowering genes expression [43]. Furthermore, *BrSOC1b* (*Bra000393*) acts with four proteins of AGAMOUS-LIKE (BrAGL9a, BrAGL9b, BrAGL2, and BrAGL8) to promote flowering in Chinese cabbage [44]. Moreover, *BcSOC1* silencing has been shown to delay bolting and stem elongation of flowering in Chinese cabbage [45]. Recently, a *B. rapa*  *OG*  *BOLTING RESISTANCE 1* (*BR1*) was identified, and its overexpression delays flowering time via vernalization and photoperiodic pathway in *Arabidopsis* [46]. These results provide valuable insights that could help genetically modulate flowering processes in *B. rapa* crops.

This study identified a novel *B. rapa*  *OG*, *BOLTING RESISTANCE 2* (*BR2)*, and investigated its effect and specific pathway that regulates flowering time in *Arabidopsis* and Chinese cabbage. Transcriptome sequencing was used to assess how *BR2* delays the flowering time in Chinese cabbage. This research aimed to provide new genetic resources and ideas for cultivating new varieties of bolting-resistant Chinese cabbage.

## Results

### Sequence analyses of *BR2* gene

*BR2* (*BraA05g000470.3C*) is a 435 bp nucleotide-long intronless gene located on *B. rapa* chromosome A05 and encodes 144 amino acids. After searching the NCBI-CDD (Conserved Domain Database) and Pfam databases, it was found that this gene does not have any structural domains. Furthermore, the BR2 protein had no signal peptide and cleavage site. The Plant TFDB database predicted that the *BR2* gene was not a transcription factor. Moreover, the GPS (Group-based Prediction System) online server revealed that BR2 protein does not have kinase activity. The predicted results indicated that *BR2* is a novel unknown functional gene, and the specific functions of *BR2* need further analysis.

### Overexpressing *BR2* delayed flowering in *Arabidopsis*

**Figure 1:**
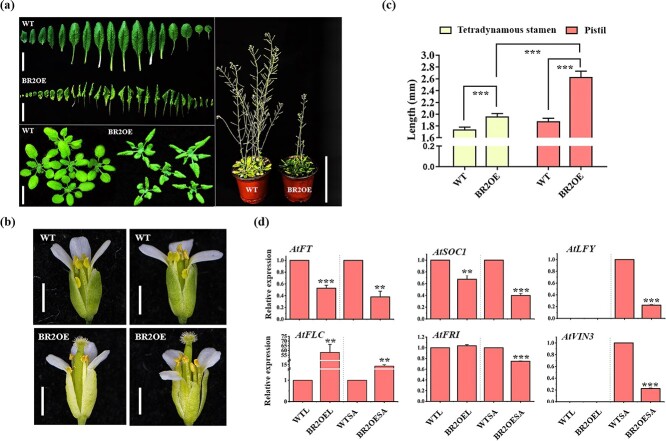
Phenotypic characterization of ‘BR2OE’ lines. **(a)** The phenotype of ‘BR2OE’ plants. The images of 28-day-old leaves, scale bars = 2 cm (left part). The images of 50-day-old plants, scale bars = 10 cm (right part). **(b)** The phenotype of WT (upper part) and ‘BR2OE’ (lower part) flowers. The stamens and pistils were observed by removing one petal. Scale bars = 1 mm. **(c)** Tetradynamous stamens and pistils length of WT and ‘BR2OE’. Data are means ± SE (*n* = 10). **(d)** Expression analysis of key flowering pathway genes in *Arabidopsis* ‘BR2OE’ and WT plants. WTL and BR2OEL represent the leaves of WT and ‘BR2OE’ plants, respectively. WTSA and BR2OESA represent shoot apex (SA) of WT and overexpression lines, respectively. The asterisk at the top of the column chart represents the significant differences detected by the Student’s *t*-test (^*^P < 0.05, ^**^P < 0.01, ^***^P < 0.001).

The flowering time of *BR2* overexpressing *Arabidopsis* plants was significantly delayed under long-day (LD) conditions, with an average delay of 33 days (Fig. 1a; Table S1, see online supplementary material). Furthermore, the leaves of the ‘BR2OE’ plants were all curled inward (Fig. 1a). The ‘BR2OE’ rosette radius, silique length, and leaves length were all shorter than the wild-type (WT) plants. The number of seeds in each silique of ‘BR2OE’ plants was significantly lower than that in WT plants. The length of tetradynamous stamens and pistils of the ‘BR2OE’ plants were all much longer than those of WT plants (Fig. 1b and c). In the ‘BR2OE’ plants, the tetradynamous stamens were lower than the pistils, while the tetradynamous stamens in WT plants were flush with or below the pistils.

To investigate whether the flowering time delay is related to the levels of its modulatory factors, the expression of some flowering key genes in the leaves and shoot apex (SA) of ‘BR2OE’ and WT plants were compared by qRT-PCR (Fig. 1d). It was revealed that the expression levels of the flowering pathway genes were similar to that observed in the delayed flowering phenotype. In the leaves and SA of the ‘BR2OE’ plant, the expressions of *AtFT* and *AtSOC1* were markedly down-regulated, and *AtLFY* in SA of the ‘BR2OE’ was similar to *AtFT* and *AtSOC1* expression. Furthermore, the *AtLFY* expression in the leaves of the ‘BR2OE’ and WT was not detected. Interestingly, *AtFLC* in leaves and SA increased by nearly 58 and 13 times compared with WT. Moreover, *AtFRI* and *AtVIN3* were notably down-regulated in SA, suggesting that *BR2* may mainly function through the vernalization pathway.

### ‘BR2OE’ was responsive to vernalization

**Figure 2:**
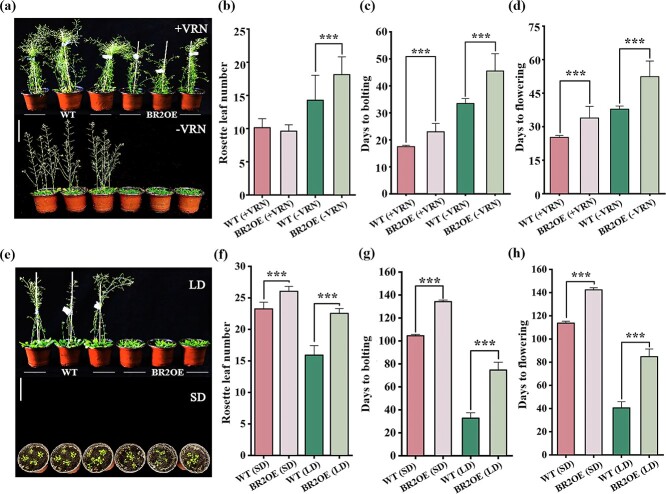
Flowering time of ‘BR2OE’ plants under different vernalization and photoperiod treatments. **(a)**–**(d)** Vernalization (+VRN) and non-vernalization (-VRN) treatments. **(a)** Above: +VRN, below: −VRN. **(e)**–**(h)** Long day (LD) and short day (SD) treatments. **(e)** Above: LD; below: SD. The photos in **(a)** and **(e)** were taken on the 46th day after sowing. Scale bars = 10 cm. Data are expressed as means ± SE. Asterisk (^*^) shows significant differences (^*^P < 0.05, ^**^P < 0.01, ^***^P < 0.001) assessed by the Student’s *t*-test. Experiments were repeated thrice (*n* ≥ 30).

The vernalized ‘BR2OE’ plants had bolting at about 23 days and flowering at about 34 days, which was much earlier than the non-vernalized plants (about 45 and 52 days); interestingly, this was still later than the WT plants in both vernalization and non-vernalization treatments (Fig. 2a, c, and d). Furthermore, the average rosette leaf number of ‘BR2OE’ plants was more than that of WT plants under non-vernalization treatment, while there was no significant difference between ‘BR2OE’ and WT plants under vernalization condition (Fig. 2b). These observations showed that ‘BR2OE’ plants positively respond to vernalization treatment.

### ‘BR2OE’ indicated late flowering under both LD and SD conditions

To assess if the late flowering phenotype of *Arabidopsis* ‘BR2OE’ plants was linked with the photoperiod pathway, the flowering time of WT and ‘BR2OE’ plants under LD and SD conditions was measured (Fig. 2c and d). The bolting and flowering times of ‘BR2OE’ plants were much later than WT plants under both LD and SD conditions. Moreover, it has been indicated that late-flowering plants form more rosette leaves when ‘BR2OE’ plants flowering under different conditions. Thus, ‘BR2OE’ plants were bolting and flowering later than WT and had more rosette leaves under LD and SD conditions. This suggests that *BR2* may not be involved in the photoperiodic pathway of flowering control.

### *BR2* gene promoter expression analysis

The *BR2* promoter sequence was cloned and constructed upstream of the GUS gene of the pCAMBIA1305.1 vector. For GUS staining, homozygous plants of *A. thaliana* were obtained by using the floral-dip method. Histochemical staining for the transgenic line’s GUS activity indicated high expression in the SA, leaves, leaf veins, stamens, stigmas, and pedicel-silique junction (Fig. S1a, see online supplementary material) but the expression of the *BR2* gene promoter was very low in siliques and seeds. These data indicated that the levels of *BR2* promoter expression are spatiotemporally regulated.

### Subcellular localization of *BR2*

The BR2 protein’s subcellular localization was assessed by injecting the tobacco leaves with a mixture of *Agrobacterium tumefaciens* carrying 35S::BR2::GFP and YFP cell membrane marker plasmids, and fluorescence was observed by confocal microscope. The distribution of fluorescence signal of the fusion protein transiently expressed in cells suggested that BR2 was present on the cell membrane (Fig. S1b, see online supplementary material).

### *BR2* expression patterns in bolting resistant type and bolting non-resistant type Chinese cabbage

**Figure 3:**
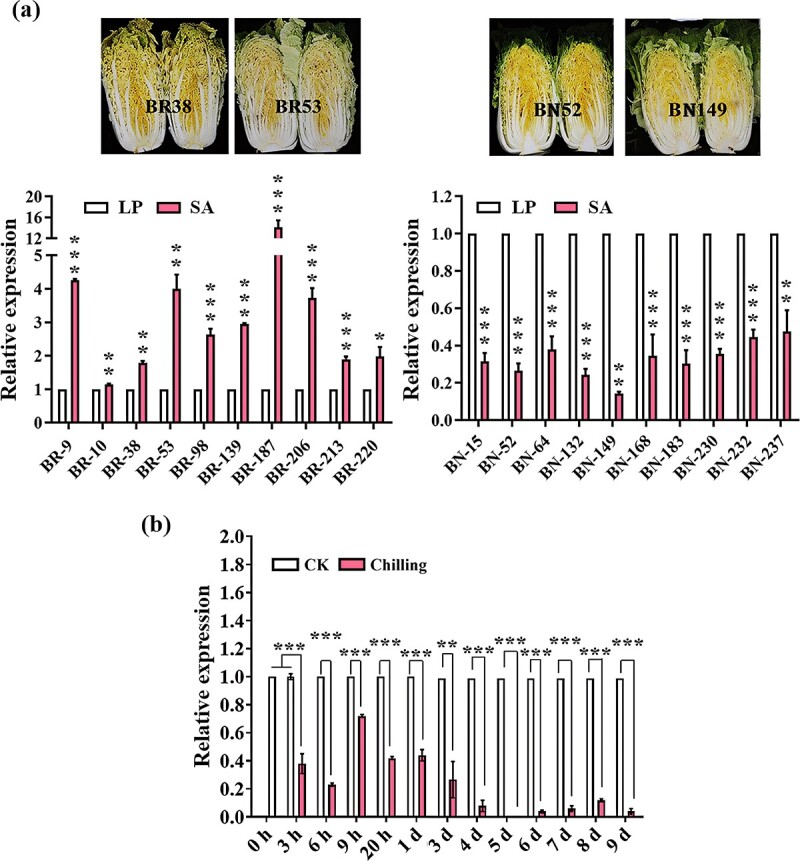
Expression of *BR2* gene in Chinese cabbage. **(a)** Phenotypes of bolting resistant type (BR type) and bolting non-resistant type (BN type) Chinese cabbage, and qRT-PCR assessment of *BR2* expression in BR and BN types at the heading stage. (LP: the top point of the inner leaf; SA: shoot apex). **(b)** The expression of *BR2* is sensitive to chilling stress in Chinese cabbage. Data are given as means ± SE of three biological replicates. The asterisk at the top of the column chart in **(a)** and **(b)** represents the significant differences detected by the Student’s *t*-test (^*^P < 0.05, ^**^P < 0.01, ^***^P < 0.001).

Because the overexpression of *BR2* showed late flowering in *Arabidopsis*, the expression of *BR2* in Chinese cabbage bolting resistant type (BR type) and bolting non-resistant type (BN type) varieties was analysed. Chinese cabbage indicating short stem with rounded apices belongs to the BR type, while the BN type has pointed apices [46, 47]. The expression pattern of *BR2* in the heading stage was analysed by selecting 10 BR type and 10 BN type varieties (Fig. 3a). The data revealed that *BR2* expression was notably increased in all BR types but was markedly down-regulated in the BN type (Fig. 3a). These results further confirmed that *BR2* was related to the bolting resistance of Chinese cabbage. Moreover, using Chinese cabbage inbred line ‘GT-24’, the expression pattern of *BR2* was analysed under chilling stress (4°C). After the treatment, *BR2* expression was substantially decreased compared with the control (Fig. 3c), which may promote vegetative growth transformation to reproductive growth.

### *BR2* overexpression in Chinese cabbage caused delayed flowering

**Figure 4:**
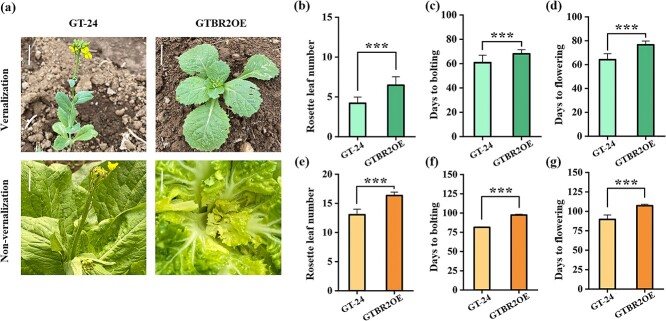
Phenotypic characterization of ‘GTBR2OE’ plants. **(a)** Phenotypic analysis of ‘GTBR2OE’ under vernalization and non-vernalization conditions. Phenotypes of 9-week-old (upper part) and 13-week-old (lower part) plants. Scale bars = 2 cm. **(b)**–**(d)** The rosette leaf number, bolting days and flowering days of ‘GT-24’ and ‘GTBR2OE’ under vernalization conditions. **(e)**–**(g)** The rosette leaf number, bolting days and flowering days of ‘GT-24’ and ‘GTBR2OE’ under non-vernalization conditions. The asterisk shows significant differences (^*^P < 0.05, ^**^P < 0.01, ^***^P < 0.001) by Student’s *t*-test. Data are depicted as means ± SE (*n* = 18).

To further assess the effect of the *BR2* on the phenotype of Chinese cabbage, T_2_ generation *BR2* overexpressing homozygous plants were obtained (Fig. S2, see online supplementary material) and referred to as ‘GTBR2OE’. The phenotypes of wild-type Chinese cabbage ‘GT-24’ and ‘GTBR2OE’ plants under vernalization and non-vernalization conditions were observed (Fig. 4a). The number of rosette leaves of ‘GTBR2OE’ plants were more than those of the ‘GT-24’ plants in both vernalization and non-vernalization conditions (Fig. 4b and e). Under vernalization conditions, the bolting and flowering times of ‘GTBR2OE’ plants (about 68 and 77 days, respectively) were later than those of the ‘GT-24’ plants (about 61 and 64 days, respectively) (Fig. 4c and d). Similarly, the bolting and flowering times of ‘GTBR2OE’ plants (about 97 and 107 days, respectively) were later than those of the ‘GT-24’ plants (about 81 and 90 days, respectively) under non-vernalization conditions (Fig. 4f and g).

The morphological traits including plant weight, head weight, plant height, plant width, outer leaf length, outer leaf width, head height, head width, and shortened stem width of ‘GT-24’ and ‘GTBR2OE’ plants during the heading stage were significantly different (Fig. S3, see online supplementary material). Overall, *BR2* regulates the bolting resistance of Chinese cabbage; however, the possible pathways need further analysis.

### Transcriptome analysis of ‘GTBR2OE’ plants

To analyse the molecular mechanisms associated with the impact of *BR2* on delayed flowering time, transcriptomic sequencing was performed by preparing the cDNA libraries from the SA of ‘GT-24’ and ‘GTBR2OE’ plants before bolting after vernalization treatment. All samples indicated Q20 and Q30 values >97% and >92%, respectively (Table S2, see online supplementary material). The raw and clean read numbers for each sample ranged from 41 500 918 to 48 549 908 and 37 384 288 to 43 933 202, respectively, consistent with highly reliable transcriptome detection results.

**Figure 5:**
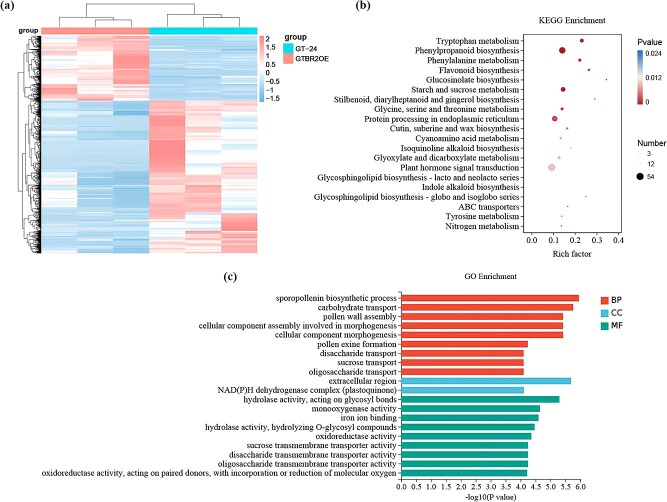
Identification of DEGs between ‘GTBR2OE’ and ‘GT-24’ plants. **(a)** Heatmap of DEGs. **(b)** The top 20 enriched KEGG pathways are associated with the identified DEGs. Pathways are provided with the corresponding rich factors. **(c)** DEG gene ontology (GO) classification was carried out, with individual bars denoting the numbers of DEGs mapped to particular GO categories. Red: biological process (BP), blue: cellular component (CC), green: molecular function (MF).

To assess the transcriptomic alterations linked with the delayed flowering time of *BR2* overexpression, each unigene’s FPKM values in the ‘GT-24’ and ‘GTBR2OE’ plants were compared to identify differentially expressed genes (DEGs). Altogether, 2864 DEGs were determined, of which 2011 (70.22%) and 853 (29.78%) were downregulated and upregulated, respectively (Fig. 5a; Table S3, see online supplementary material). KEGG pathway enrichment analysis identified 20 significantly enriched KEGG pathways for each DEG (Fig. 5b; Table S4, see online supplementary material). These DEGs were primarily enriched in pathways of ‘tryptophan metabolism (brp00380)’, ‘phenylpropanoid biosynthesis (brp00940)’, ‘phenylalanine metabolism (brp00360)’, ‘flavonoid biosynthesis (brp00941)’, ‘glucosinolate biosynthesis (brp00966)’, ‘starch and sucrose metabolism (brp00500)’, ‘stilbenoid, diarylheptanoid and gingerol biosynthesis (brp00945)’, ‘glycine, serine and threonine metabolism (brp00260)’, ‘protein processing in endoplasmic reticulum (brp04141)’, and ‘plant hormone signal transduction (brp04075)’. Furthermore, 20 significantly enriched GO terms were also identified *via* GO enrichment analysis of these DEGs (Fig. 5c;  Table S5, see online supplementary material). These included primarily enriched biological processes (including sporopollenin biosynthetic process, carbohydrate transport, pollen wall assembly, cellular component assembly involved in morphogenesis, cellular component morphogenesis, and pollen exine formation), cellular components (including extracellular region, NAD(P)H dehydrogenase complex (plastoquinone)), and molecular function (including hydrolase activity, monooxygenase activity, iron ion binding, oxidoreductase activity, sucrose transmembrane transporter activity, disaccharide transmembrane transporter activity, oligosaccharide transmembrane transporter activity, and oxidoreductase activity) terms.

**Figure 6:**
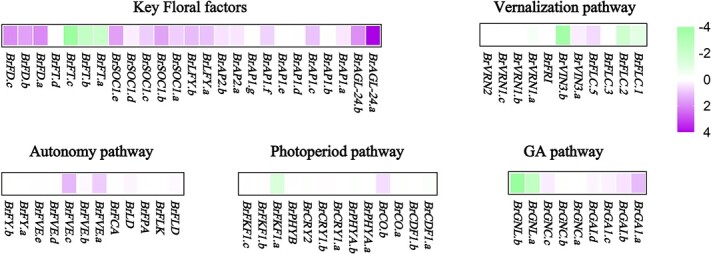
Heatmap of flowering genes. Heatmap composed of log2 fold-change values of several flowering genes under different pathways. GA pathway represented the gibberellin pathway.

To investigate the dynamic expression changes of the main flowering related genes in ‘GTBR2OE’ compared to ‘GT-24’, plants such as *BrFLCs*, *BrFTs*, *BrVIN3s*, *BrSOC1s*, *BrFRI*, *BrLFYs*, etc. were analysed via the heatmap. The results revealed that the expression levels of many key floral factors significantly increased, which was consistent with the phenotype of immediate bolting (Fig. 6; Table S6, see online supplementary material). However, among the vernalization pathway, the expression of most related genes was significantly inhibited, especially *BrFLC5*, which indicates that ‘GTBR2OE’ has stronger bolting resistance than ‘GT-24’ plants after vernalization treatment. The expression of genes related to other flowering pathways mostly did not show significant changes or expressions were inhibited.

**Figure 7:**
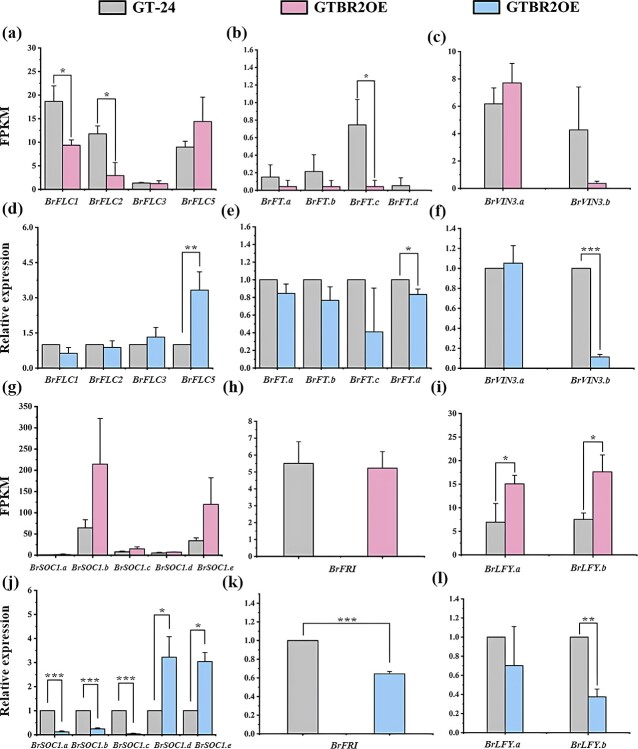
Transcriptomic sequencing data verification *via* qRT-PCR. Expression of **(a)**  *BrFLCs*, **(b)**  *BrFTs*, and **(c)**  *BrVIN3s* in transcriptomic sequencing results. Expression analysis of **(d)**  *BrFLCs*, **(e)**  *BrFTs*, and **(f)**  *BrVIN3s via* qRT-PCR. Expression of **(g)**  *BrSOC1s*, **(h)**  *BrFRI*, and **(i)**  *BrLFYs* in transcriptomic sequencing results. Expression of **(j)**  *BrSOC1s*, **(k)**  *BrFRI*, and **(l)**  *BrLFYs via* qRT-PCR. For qRT-PCR, each value is given as the means ± SE (*n* = 3), for transcriptomic sequencing, each value is the mean of three replicates. The asterisk (*) shows significant differences (^*^P < 0.05, ^**^P < 0.01, ^***^P < 0.001) by Student’s *t*-test.

To verify the accuracy of transcriptome sequencing data, the expression of a few critical flowering genes was verified by qRT-PCR (Fig. 7). Compared to the ‘GT-24’ controls, the ‘GTBR2OE’ plants indicated markedly up-regulated *BrFLC5* expression (Fig. 7a and d), while all four homologous genes of *BrFT* showed decreased expressions (Fig. 7b and e), consistent with the delayed flowering phenotype. Furthermore, qRT-PCR indicated that *BrVIN3.b* of two *BrVIN3* homologous genes (Fig. 7c and f), *BrSOC1.a*, *BrSOC1.b*, and *BrSOC1.c* of five *BrSOC1* homologous genes (Fig. 7g and j), and *BrFRI* gene (Fig. 7h and k), and *BrLFY.b* (Fig. 7l) were significantly downregulated. These results indicated that the *BR2* gene may regulate flowering primarily through the vernalization pathway, which further supported a model in which *BR2* serves as an important regulator of the floral transition in Chinese cabbage.

## Discussion

The *OGs* are crucial for species formation and evolution as they regulate mechanisms for generating new genes and functions [48]. Furthermore, they exist in almost every genome and are associated with various biological pathways, such as immune regulation, species-specific adaptation processes, metabolism, stress biology, and species-specific traits regulation [49]. However, most *OGs* are not well annotated because they lack identifiable functional domains, making their functional characterization difficult [1]. The functional characterization of *OGs* is essential, which helps us understand the functional diversity of the genome and the evolution of complex traits [49]. Here, an *OG*, *BR2* was identified, which positively regulates bolting resistance and delayed flowering. Since the differentiation of Chinese cabbage from *Arabidopsis*, specific traits have been observed such as leafy head, and Chinese cabbage requires vernalization to enter reproductive growth. *BR2* overexpression delays flowering, which further confirms the relationship between OGs and the formation of species-specific traits. *BR2* also regulates bolting resistance, as previously reported [46]. In *Arabidopsis*, *BR1* overexpression markedly delays the flowering time. Furthermore, it has been verified that the nuclear BR1 protein acts via the photoperiod and vernalization pathways [46]. *A. thaliana* species-specific *OG*  *Qua-Quine Starch* (*QQS*) specifically modulates nitrogen and carbon allocation, decreases starch, and increases protein [5]. Moreover, *QQS* has shown similar roles across species, including *Arabidopsis*, soybean, tobacco, and others [3, 4, 12]. The protein–protein interactions analysis of NF-YC4, a QQS interaction protein, indicated that many proteins are a part of the transcription factor NF-Y complex, three of which are linked with flowering time [12]. Previous study showed that an *OG*  *Prod1* was necessary for preaxial digit formation during salamander limb development, which provided new insights into species-specific traits regulation of *OGs* [50]. The analysis of *OGs* in the Cucurbitaceae genomes indicated that many *OGs* are involved in reproductive growth and development processes [21]. *Brassica*-specific *OG*  *BrLFM* is proved to be involved in leafy head formation in Chinese cabbage, which will be vital for understanding the relationship between *OGs* and species-specific morphological features, such as the leafy head in Chinese cabbage [51]. These data validate the crucial role of *OGs* in the regulation of species-specific traits, providing a theoretical basis for the comprehensive understanding of *BR2*.

Floral transition requires an appropriate time [24]. Previous studies have revealed that the regulatory network of flowering time includes vernalization and another four pathways [52–54]. In Chinese cabbage, vernalization is necessary for the reproductive development to bolting and flowering [55]. The ‘BR2OE’ plant is a late flowering phenotype of *Arabidopsis*, which revealed that *BR2* regulates flowering time. ‘BR2OE’ indicated late flowering under both LD and SD conditions, while ‘BR2OE’ was responsive to vernalization, indicating that *BR2* may be not involved in the photoperiodic pathway of flowering control and probably regulates flowering time via the vernalization pathway. Similarly, an *Arabidopsis* glycosyltransferase gene *UGT87A2* regulates flowering time via the flowering repressor *AtFLC*, and *UGT87A2* is not involved in the photoperiod pathway of flowering control [56]. Furthermore, *AtFLC* expression was markedly reduced in ‘BR2OE’ (Fig. 1d), which is consistent with data on leaf quantity. Increased *AtFLC* levels delay flowering time by reducing the transcripts of *AtFT* and *AtSOC1*. Here, it was identified that *BR2* positively modulates the expression of *FLC*, which delays flowering in *Arabidopsis* ‘BR2OE’ plants. Moreover, *BR2* overexpression in Chinese cabbage also indicated a bolting resistance phenotype, consistent with the phenotyptic analysis of ‘BR2OE’ plants. The decreased expression of some flowering integrator genes including three *BrSOC1s*, two *BrLFYs*, and four *BrFTs*, were related to flowering delays in ‘GTBR2OE’ plants. It is worth noting that 14 of 18 genes (77.78%) verified transcriptomic sequencing results via qRT-PCR have consistent expression trends, which demonstrates the accuracy of the experimental results. The literature has indicated that the downregulation of floral integration factors is directly linked with flowering delay [44, 45, 57], consistent with the results of this research. The qRT-PCR and transcriptomic sequencing confirmed that *BR2* overexpression in *B. rapa* markedly altered *BrFLC5* expression and down-regulated key floral genes. After vernalization, ‘GTBR2OE’ still has stronger bolting resistance than ‘GT-24’ plants. These results indicate that the role of *BR2* in the regulation of bolting resistance may involve the vernalization pathway. In *B. rapa* vegetables, flowering time is essential trait and is modulated by *FLC* [58]. A recent study indicated that when overexpressed *BrFLC5*, which acts as a floral repressor in *A. thaliana*, and its increased expression promotes vernalization and might help develop an enhanced bolting-resistant breed of *B. rapa* vegetables [59]. Another study showed that among the three analysed *BrFLCs*, *BrFLC5* had the lowest expression, suggesting that it might be a weak flowering time modulator in *B. rapa*; however, it might be more efficient for breeding premature bolting resistance in *B. rapa* [43]. Furthermore, *BrFLC5* has also been observed as a crucial candidate gene for a flowering time control in *B. rapa* [38, 60]. The downregulation of *BrVIN3.b* expression, a direct inhibitor of *FLC*, explained the increased expressions of *BrFLC5*. Studies showed that the mutations of *AtVIN3* results in a vernalization-insensitive phenotype [28]. These results strongly support the findings of this study.

Based on the aforementioned data, it can be concluded that *BR2* modulates flowering time via the vernalization pathway. Since *BR2* is localized on the cell membrane, its downstream interaction proteins and upstream regulatory TFs can be identified through yeast two-hybrid (Y2H) screens, and yeast one-hybrid (Y1H) screens, respectively. Furthermore, *BR2* knockout in Chinese cabbage via CRISPR/Cas9 fully characterized how *BR2* regulates bolting resistance. Most protein-coding *OGs* seem to be generated from non-coding sequences, and some *OGs* will continue to exist throughout the long evolutionary process [61], thus, the evolutionary origin and the exact mechanisms of *BR2* warrant additional in-depth research. These analyses jointly identified a new *OG*, *BR2*, a key regulatory factor for bolting resistance in Chinese cabbage. This investigation provided novel ideas and gene targets for the cultivation of new bolting-resistant varieties of Chinese cabbage, which will offer valuable insights into the functions of *OGs* in the regulation of species-specific traits.

## Materials and methods

### Plant materials and cultivation

Plant materials include Chinese cabbage inbred line ‘GT-24’ and ‘Chiffu’, wild-type *A. thaliana* ‘Col-0’ (WT) and Arabidopsis *BR2* overexpression plant ‘BR2OE’, Chinese cabbage *BR2* overexpression plants ‘GTBR2OE’ and *Nicotiana benthamiana*. The two varieties of Chinese cabbage (BR and BN types) were cultivated in our laboratory (Molecular Biology of Vegetable Laboratory, College of Horticulture, Shenyang Agricultural University). *N. benthamiana* was cultivated as in prior studies [46]. The plants were grown in conditions described in previous studies [46]. Plants were grown under long-day (LD, 16 h light/8 h darkness), and short-day (SD, 8 h light/16 h darkness) conditions.

### *BR2* sequence analyses, vector construction and plant transformation

The analyses of the *BR2* sequence were performed as detailed previously [46]. For *BR2* overexpression in *Arabidopsis* and Chinese cabbage, vector construction, plant transformation and selection, DNA isolation, and PCR amplification for positive transgenic plants detection were carried out as detailed in prior reports [23, 46, 62]. Primer pairs utilized are listed in Table S7 (see online supplementary material).

### Vernalization and LD/SD treatments

For vernalization treatment, the seed sprouted WT and ‘BR2OE’ plants were grown at 4°C for 6 weeks, and then transferred to LD conditions under normal growth temperature. For LD/SD treatments, WT and ‘BR2OE’ plants were grown in LD or SD conditions. The investigation of rosette leaf number, days to bolting and flowering of WT as well as ‘BR2OE’ plants were measured and compared at the same developmental stage by following previous methods [46]. At least 30 plants were used for each experiment.

### Histochemical GUS assay and subcellular localization analyses

Histochemical GUS assay was performed as described in previous methods [62]. The subcellular localization analyses of the BR2 protein were carried out by referring to previous research [46]. The cell membrane was observed by assessing its marker, YFP. All assays were repeated thrice. Cells were observed using the Leica confocal microscope (Wetzlar, Germany) after 48 h of agro-infiltration. Primer pairs utilized for vector construction are provided in Table S7 (see online supplementary material).

### Analyses of *BR2* expressions in Chinese cabbage

The expressions of the *BR2* in Chinese cabbage BR and BN type varieties were analysed by following previous research [46]. The top point of the inner leaf (LP) and shoot apex (SA) were sampled, and LP was set as a control. There were three biological replicates and in each replicate, three plants of different lines were sampled. For chilling stress in Chinese cabbage, seeds of Chinese cabbage inbred line ‘GT-24’ were maintained at 25°C in LD condition for 2 days of germination treatment, then transferred to 4°C for different times ranging from 0 to 9 days. These plants were called the chilling group, which together with the control group (CK) was incubated at 25°C. There were three biological replicates and in each replicate, 10 seedlings from different times were sampled. The collected samples were snap-frozen using liquid nitrogen and then stored at −80°C for RNA isolation. qRT-PCR primers employed for gene expression analyses are given in Table S7 (see online supplementary material).

### Phenotypic investigation

For phenotypic investigation of *Arabidopsis* ‘BR2OE’ plants, previous research was referred to [46]. For phenotypic investigation of Chinese cabbage ‘GTBR2OE’ plants under vernalization and non-vernalization conditions, after 2 days of initial germination, ‘GT-24’ and ‘GTBR2OE’ seeds were then germinated at 4°C for 30 days, transferred to the plug tray, and planted in soil on 10 April 2022. The non-vernalization seeds were directly planted in the plug tray on 25 March 2022, and planted in the soil after 30 days of cultivation. Upon first flower bud appearance, the number of bolting days and rosette leaf numbers were recorded; upon first flower appearance, the days to flowering were recorded.

For phenotypic analysis of Chinese cabbage ‘GTBR2OE’ plants under heading stage, the seeds of ‘GT-24’ and ‘GTBR2OE’ were directly sown in the plug tray for 30 days before plantation in the field. The mature plants of ‘GT-24’ and ‘GTBR2OE’ were selected during the harvest stage for the observation of heading traits. Outer leaves numbers were defined as the number of leaves outside the compact spherical shape. Plant weight was the weight of the entire plant, while the head weight was the weight of the leafy head without the external leaves and roots. Furthermore, plant height and width were the measures of the height and width of the whole plant, head height and width were the measures of the height and width of the leafy head. Moreover, outer leaf length and width were the measures of the length and width of outermost leaves, while shortened stem height and width were defined as the height and width of the shortened stem. The phenotypic observation was carried out by using stable homozygous T_2_ transgenic plants, and three lines of transgenic plants were investigated, each with at least nine plants.

### Total RNA isolated, first-strand cDNA synthesis, and qRT-PCR

These experiments were performed according to the method described in previous studies [23, 46]. All primers utilized in qRT-PCR are listed in Table S7 (see online supplementary material).

### Transcriptomic sequencing and validation

The seeds of ‘GT-24’ and ‘GTBR2OE’ were kept at 4°C for 30 days, incubated for 2 days after germination, and then planted in the soil. There were three biological replicates of shoot apex and 12 samples were obtained in each replicate, sampled from the ‘GT-24’ and ‘GTBR2OE’ plants before bolting. The collected samples were snap-frozen using liquid nitrogen and then stored at −80°C. Transcriptomic sequencing and analysis were carried out per previous research [46]. The comparison of the clean reads with the *B. rapa.* v1.5 genome was downloaded from the *Brassica* Database (BRAD) (https://brassicadb.cn/#/). qRT-PCR was carried out to validate gene expression, and there were three biological replicates. The primer pairs employed are presented in Table S7 (see online supplementary material).

### Statistical analysis

Statistical analyses were carried out using SPSS 19.0 software (Chicago, IL, USA) through Student’s *t*-test, as described in the literature [47]. The data are displayed as means ± SE.

## Supplementary Material

Web_Material_uhae216

## Data Availability

The transcriptome data in this study have been deposited in the Sequence Read Archive (SRA) database in NCBI, with bioProject accession number PRJNA1073421 (https://www.ncbi.nlm.nih.gov/sra/PRJNA1073421).
