# Supplementary material for: Orphan gene BR2 positively regulates bolting resistance through the vernalization pathway in Chinese cabbage
Source: Hortic Res. 2024 Jul 30;11(10):uhae216. doi: 10.1093/hr/uhae216 (PMC11469923; doi:10.1093/hr/uhae216)
Supplement: Web_Material_uhae216 [file web_material_uhae216.zip › Supplementary Data Figure.docx]

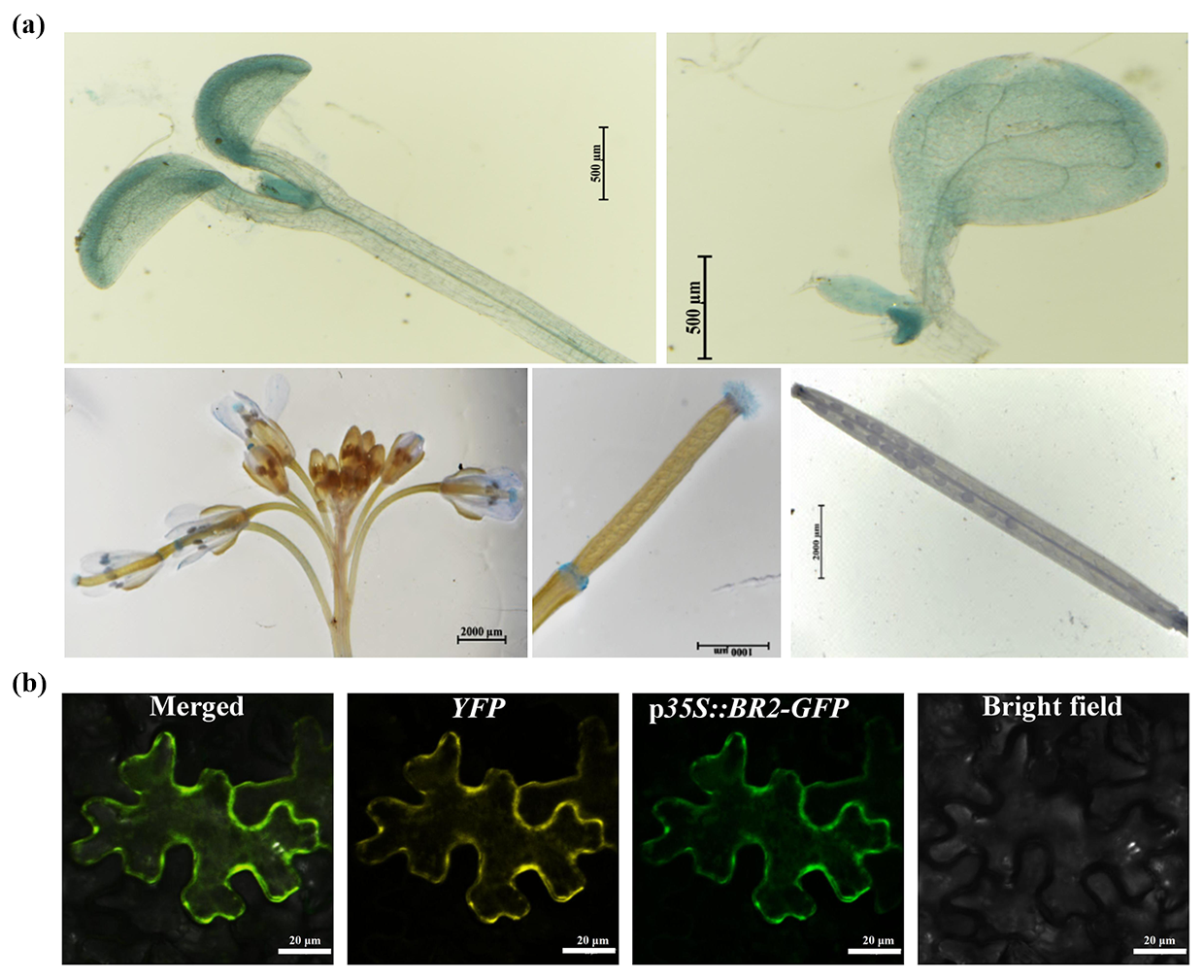


**Supplementary Data Fig. S1.** Expression analysis of *BR2* gene promoter and subcellular localization of BR2. (a) The expression analysis results of promoter fusion GUS. All scale bars were marked in the figures. (b) Subcellular location of BR2. YFP was a cell membrane marker. Scale bars = 20 μm.


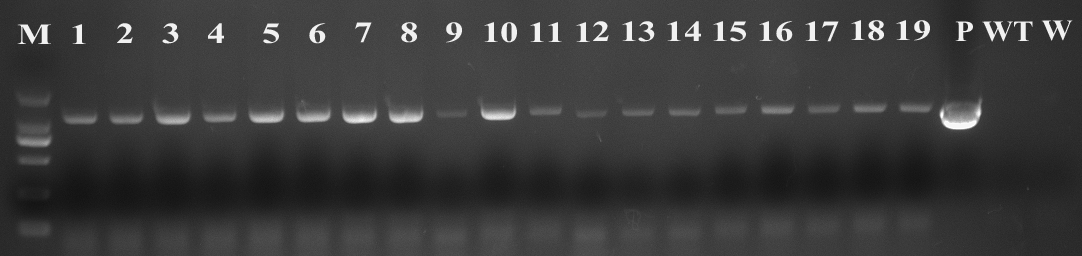


**Supplementary Data Fig. S2.** Identification of the transgenic lines by PCR. The DNA amplification of transgenic-positive plants was performed using hygromycin primers (*hyg*-F and *hyg*-R) *via* PCR. (1-19: 19 homozygous ‘GTBR2OE’ lines of T_2_ generation; P: plasmid, positive control, WT: ‘GT24’; W: water, negative control). M represented DNA marker, the band sizes from bottom to top are 100 bp, 250 bp, 500 bp, 750 bp, 1000 bp, and 2000 bp respectively.


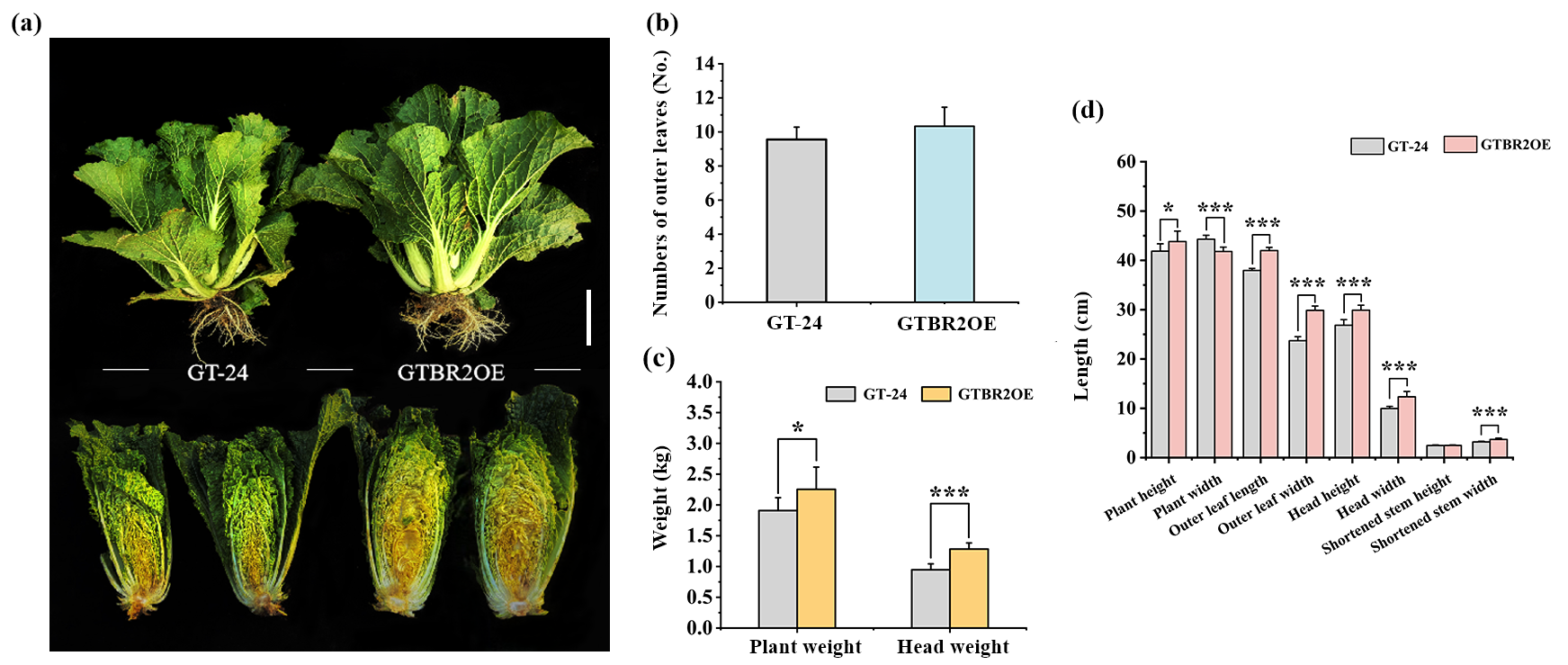


**Supplementary Data Fig. S3.** Phenotype of ‘GTBR2OE’ plants compared to ‘GT-24’ at the heading stage. (a) Phenotyptic investigations of ‘GT-24’ and ‘GTBR2OE’ plants. Scale bar = 10 cm. (b-d) Phenotypic investigations of heading traits. The asterisk (*****) shows significant differences (**p* < 0.05, ***p* < 0.01, ****p* < 0.001) by Student’s *t*-test. Data are presented as means ± SE (n = 9).
